# Tissue chips as headway model and incitement technology

**DOI:** 10.1016/j.synbio.2024.08.007

**Published:** 2024-08-30

**Authors:** Prerna Suchitan Modi, Abhishek Singh, Awyang Chaturvedi, Shailly Agarwal, Raghav Dutta, Ranu Nayak, Alok Kumar Singh

**Affiliations:** a Amity Institute of Biotechnology, Amity University Uttar Pradesh, Noida, Uttar Pradesh, India; b Amity Institute of Nanotechnology, Amity University Uttar Pradesh, Noida, Uttar Pradesh, India

**Keywords:** 3D cell culture, Microfluidic device, Organ-on-chip, 2D cell culture, 3-Dimensional spheroid

## Abstract

Tissue on a chip or organ-on-chip (OOC) is a technology that's dignified to form a transformation in drug discovery through the use of advanced platforms. These are 3D in*-vitro* cell culture models that mimic micro-environment of human organs or tissues on artificial microstructures built on a portable microfluidic chip without involving sacrificial humans or animals.

This review article aims to offer readers a thorough and insightful understanding of technology. It begins with an in-depth understanding of chip design and instrumentation, underlining its pivotal role and the imperative need for its development in the modern scientific landscape. The review article explores into the myriad applications of OOC technology, showcasing its transformative impact on fields such as radiobiology, drug discovery and screening, and its pioneering use in space research. In addition to highlighting these diverse applications, the article provides a critical analysis of the current challenges that OOC technology faces. It examines both the biological and technical limitations that hinder its progress and efficacy and discusses the potential advancements and innovations that could drive the OOC technology forward. Through this comprehensive review, readers will gain a deep appreciation of the significance, capabilities, and evolving landscape of OOC technology.

## 1 Introduction

Traditional drug development involves several phases of development including animal model testing and human trials which are not only time consuming but also incurs a huge cost, especially when trials fail at any stages of development.

Today, organ-on-chip (OOC) is recognized as a top emerging technology by the World Economic Forum, revolutionizing medical research by mimicking human organs on a small scale. The National Institute of Health (NIH) supports OOC research to address high drug failure rates in clinical phases. Recent advancements allow the scientific community to better understand human physiology, drug discovery, and disease by mimicking *in-vivo* environments. While 2D cell cultures are common, they have limitations, leading researchers to develop 3D cultures that better reflect *in-vivo* conditions.

**Fig. 1:**
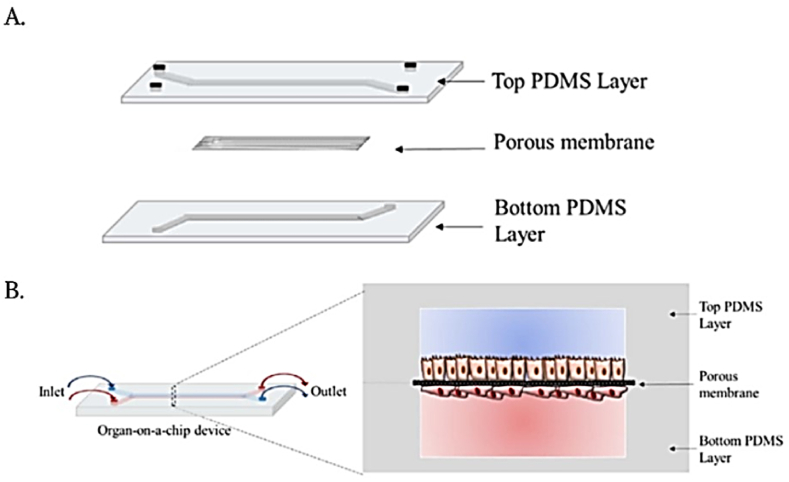
Model of a microfluidic chip with cells grown on a porous membrane. (A) A schematic representation of a two-layered device with porous membranes. (B) Microfluidic device containing endothelial cells on the basolateral side of the membrane and organ-specific cells on the apical side (PDMS). *Adapted with permission from ref.* [31].

Microfluidic devices have been used for almost 20 years for cell culture. However, prior to the development of multi-compartment organ chips, cells were simply cultured on conventional, rigid substrates placed within a single flow channel [1]. Eventually, these single-channel microfluidic culture devices, and more complex ones incorporating different types of cells with or without extracellular matrix (ECM) gels, were developed as ‘multi-physiological systems’ for modelling tissue pathophysiology; these systems are also sometimes referred to as organ chips [1]. In 3D cultures, the extracellular matrix provides an organized system for cell-to-cell interactions facilitating development of dynamic tissue structures, unlike 2D cultures which do not reflect actual *in-vivo* conditions [2,3]. In organ-on-chip applications, selecting appropriate scaffolding materials based on specific cell types are crucial to mimic *in-vivo* microenvironments more closely. These materials in 3D culture systems could function as an extracellular matrix to assist in modulation of cell behaviour and development [4,5]. Materials like hydrogels offer controlled porosity that promotes an efficient flow of nutrients and oxygen. It has been demonstrated that natural and synthetic hydrogels have the same physical, chemical, and biological characteristics as ECM environments, including biocompatibility, biodegradability, and biochemical functionality. It is not possible to simulate real *in-vivo* conditions in two-dimensional cultures where cells interact intricately as tissues, exerting mechanical pressure on each other [6]. Researchers have identified specific gravity-dependent cellular biochemical interactions through large-scale genomics and proteomics studies [7]. Gravity affects key molecules controlling cellular processes like growth, division, and migration. However, these effects could not be properly investigated in 2D cell cultures. This drawback could be addressed by incorporating 3D bioprinting techniques while designing OOC platforms to provide realistic microfluidic systems mimicking the true physiology of an organ [8,9]. The advent of 3D culturing technologies has led to improvements in cell research, resulting in more accurate description of cellular processes (Fig. 1) [10]. Moreover, in the case of diseases like cancer where current models fail to provide accurate predictions of the outcomes of various clinical treatments, OOC show potential to facilitate the assessment of mechanistic determinants [[11], [12], [13], [14], [15], [16]]. In conventional animal model studies, due to apparent inter-species differences, toxicity studies on animal models may inaccurately reflect toxicological effects in humans [[17], [18], [19]]. With recent advances in microfluidics, OOC platforms have been created that combine advanced 3D tissue engineering constructs with microfluidic networks to eliminate the shortcomings of *in-vitro* 2D models [19,20].

As the demand for more realistic *in-vitro* cell culture models has increased in recent years, OOCs have been developed to meet this need, introducing microfluidics, mechanical stretch, and other physiological stimuli to *in-vitro* models, thereby significantly enhancing their descriptive abilities [21]. The development of biosensors has been facilitated by the foundation and recent advancements in mechanical analysis techniques, spanning from cellular to tissue length scales, allowing their integration into OOCs for the improvement of drug screening, disease modelling, and characterization of biological dynamics in tissue fate and function [[22], [23], [24]]. Sensors with multiplex advantages are now within reach, and additive manufacturing approaches open up new possibilities for sensor design. Despite the demonstrated benefits of incorporating mechanical cues into these models, there are relatively few methods available for measuring mechanical tissue features directly on the chip in microfluidics [22]. In OOCs, mimicking cell environments proves beneficial for generating accurate data, it requires intricate assessment of membrane material properties utilized to form the chip. Properties like stiffness, surface roughness, shape, and microstructure of membrane materials are known to affect cell growth and adherence [25]. The application of microfabrication has allowed the creation of a range of porous, micro-structured membranes that serve as an accurate representation of native tissue morphology that can be controlled and represented physiologically. Following recent developments in stem cells and genetic engineering, reliable data and information have surfaced that can be adapted towards 3D tissue fabrication, potentially transforming the paradigm in disease modelling and the study of unknown disease mechanisms, which is crucial for precision medicine [4,21,23]. The development of 3D tissues and organs will ultimately improve diagnosis and treatment due to significant advancements in materials, micro-scale technology, and stem cell biology. However, this is just the early stage of a technology that has already introduced numerous advantages. Every significant advancement faces several challenges, and similarly, the technology of OOC must undergo substantial improvements to fully demonstrate its potential benefits.

This review article aims to provide readers with a comprehensive understanding of OOC technology. It includes a fundamental description of the technology's origin, importance, and necessity. The article explores various applications of OOC technology, such as radiobiology, drug discovery and screening, and its applications in space research. Furthermore, it also addresses the current challenges faced by this technology, including both biological and technical limitations, and briefly discusses the future prospects of OOC technology.

## 2 What are tissue chips?

Tissue chips are microfluidic bioengineered 3D devices lined with living human cells that mimic the function and diversity of human organs. The tissue chips or micro-physiological systems (MPS) [26] are the smallest functional unit that represents the biochemical, functional, and mechanical microenvironment that cells experience in our bodies [27].

Materials like PDMS (poly dimethyl sulfoxide) and plastics (e.g. poly(methyl methacrylate) (PMMA) commonly known as its trade name Plexiglass) have been the primary substrate materials for most OOC platforms. They maintain a stable fluid connection without harming the cellular microenvironment components and are essentially used to manufacture chip systems [28] The chips fabricated with such material provide support due to their flexible nature and channel flow network, which facilitate nutrient and oxygen uptake by the cells. Hydrogel materials, specifically, are well-suited for mimicking native extracellular matrices (ECMs) and are frequently combined with other substrate materials to create hybrid chips. Other materials like polycarbonate (PC), polystyrene (PS), Cyclic Olefin Polymer (COP) and Cyclic Olefin Copolymer (COC) are also used as they provide applications specific mechanical properties, biodegradation rate, and biocompatibility [29].

As shown in Fig. 1, the basic design of the OOCs consists of one compartment, used to mimic the blood vessels and the other compartment(s) for the actual tissue cells [30]. The chips are equipped with two inlets and outlets for the fluid to enter and exit, as well as for the introduction of biological materials such as basal laminar proteins, cells, and therapeutic drugs [30,31] Other component is the porous membrane, whose porosity is characterized by polymeric, flat microstructures that are used to recreate the permeability between two environments, thereby facilitating cell adhesion and separation as well as communicating between the two compartments [30,32].

**Fig. 2:**
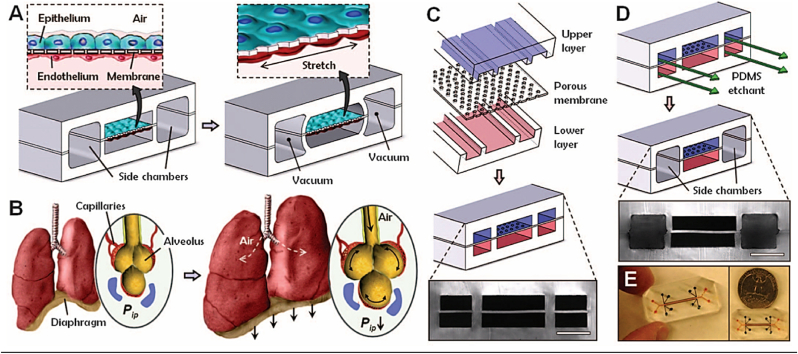
Biologically inspired design of a human breathing lung-on-a-chip microdevice. (A) The microfabricated lung mimic device uses compartmentalized PDMS microchannels to form an alveolar-capillary barrier on a thin, porous, flexible PDMS membrane coated with ECM. The device recreates physiological breathing movements by applying vacuum to the side chambers and causing mechanical stretching of the PDMS membrane forming the alveolar-capillary barrier. (B) During inhalation in the living lung, contraction of the diaphragm causes a reduction in intra-pleural pressure (Pip), leading to distension of the alveoli and physical stretching of the alveolar-capillary interface. (C) Three PDMS layers are aligned and irreversibly bonded to form two sets of three parallel microchannels separated by a 10 μm-thick PDMS membrane containing an array of through-holes with an effective diameter of 10 μm. Scale bar, 200 μm. (D) After permanent bonding, PDMS etchant is flowed through the side channels. Selective etching of the membrane layers in these channels produces two large side chambers to which vacuum is applied to cause mechanical stretching. Scale bar, 200 μm. (E) Images of an actual lung-on-a-chip microfluidic device viewed from above. *Adapted with permission from ref.* [6].

In case of lung-on-a-chip as shown in Fig. 2, the chip contains three fluidic channels with separate parenchymal and vascular compartments; in the centre it has a porous ultra-thin flexible membrane on which human cells are added which acts like air blood barrier and underneath there are capillary cells [6] The mechanical forces such as contraction and relaxation are applied to the cells sideways with the aid of vacuum channels [32] Air flows through the top of the channel and so a liquid is flowed that contains nutrients through the blood channel [27].

**Fig. 3:**
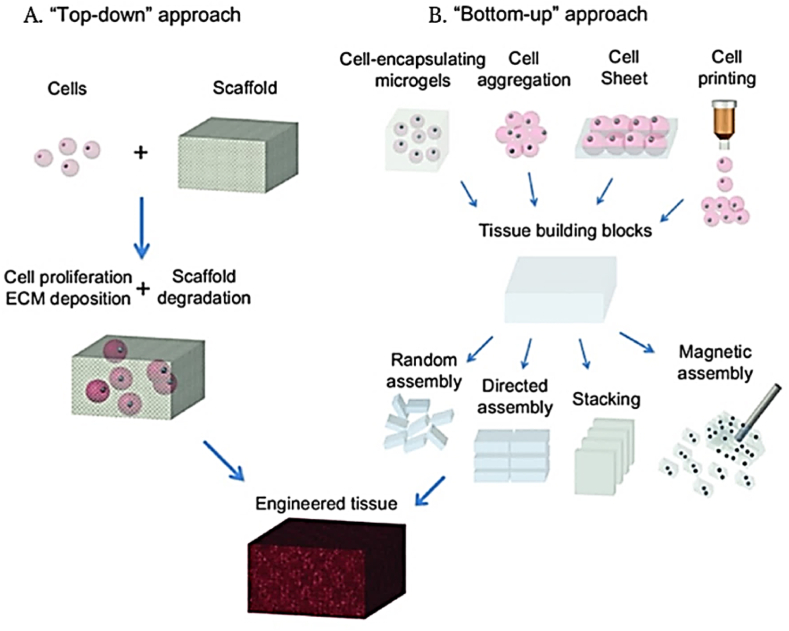
Schematic of "top-down" and "bottom-up" approaches for tissue engineering. (A) In the top-down approach, cells are seeded on a biocompatible and biodegradable scaffold and are expected to populate in the scaffold and create their own extracellular matrix. (B) In the bottom-up approach, various methods are utilized for generating tissue building blocks and these units can be engineered into large tissue constructs via multiple assembling methods [34].

Engineering of lung tissue begins with the isolation of cells from the human body which are genetically reprogrammed into induced pluripotent stem cells (iPSCs). These cells have the ability to differentiate into organ-specific cells [23] The cells are then provided with the adequate growth medium for its survival and native environment to experience the same physical stress and strain for its proper functioning [33] Several thin or avascular tissues have been engineered successfully *in-vitro* with the aid of the top-down or bottom-up approach based on biomimetic scaffolds. The traditional, top-down approach involves seeding cells into full sized porous scaffolds to form tissue constructs [34] This approach poses many limitations such as slow vascularization, diffusion limitations, low cell density and non-uniform cell distribution. In contrast, the modular or bottom-up approach involves assembling small, non-diffusion limited, cell-laden modules to form larger structures and has the potential to eliminate the shortcomings of the traditional approach (Fig. 3) [34].

The architecture of microfluidic channels is aimed to provide tissue with vasculature to bring in blood and nutrients and to take away the wastes, mimicking human vasculature [34]The evolvement and improvement of tissue chip platforms in device designing, incorporating different cell types, and integrating different platforms, relies heavily on the cell type(s) used and their response to culture conditions provided within the device [32,35,36].

Single OOC systems revolutionized biomedical research by emulating the intricate functionalities of individual organs *in-vitro*. For instance, heart OOCs, constructed to replicate contractility and electrical activity, utilize induced pluripotent stem cells (iPSCs) to assess beat rate, force generation, and excitation thresholds. Similarly, lung OOCs, facilitated studies on pulmonary drug absorption while brain OOCs provided insights into selective drug penetration and neurovascular interactions. These systems represented versatile tools with diverse applications ranging from drug screening and disease modelling to personalised medicine and fundamental physiological research [37].

Yet, the true power of this technology really comes from the fact that they can be connected or linked fluidically to form a virtual human on a chip for better predictions from its functionality with the exposure of certain drugs. Various studies have developed multi-Organ-on-a-Chip (multi-OoC) systems that support organ-organ communication or crosstalk. This allows for modelling of systemic diseases and the study of multiorgan processes. These platforms could also aid patient-specific disease modelling and treatment development of rare diseases [38]. Some of the multi-OoC include systems with gut and liver co-culture [39,40], liver and kidney co-culture [41,42], liver and heart co-culture [43,44], and many other [[45], [46], [47], [48]]. Recent advancements in OOC technology have led to the development of platforms integrated with sensors, enabling real-time monitoring of various parameters crucial for evaluating drug responses over extended periods [37].

## 3 Applications of tissue chips

**Fig. 4:**
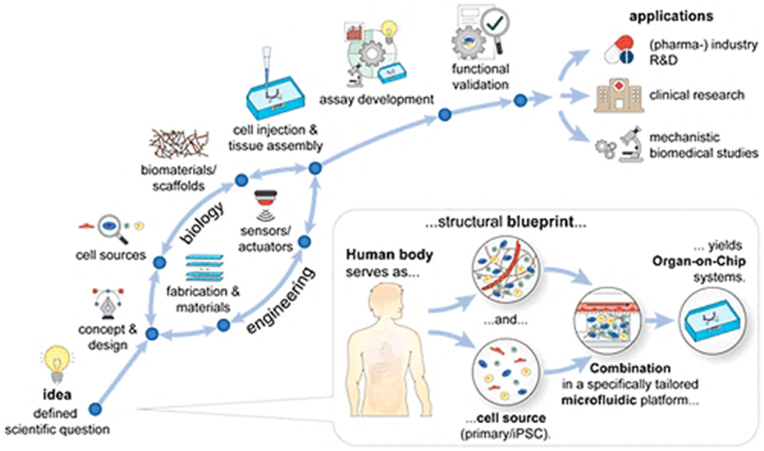
A schematic depiction of the origins and development of organs-on-chips *Adapted with permission from ref.* [55].

Considering the progress and utilization of tissue chips or micro-physiological systems (MPS), these have been in usage since 2012 by many pharmaceutical companies and laboratories [49]In the forthcoming groundwork, scientists are working on production of different kinds of specific organ chips namely, liver, gut, lungs, heart, bone marrow, kidney, brain, intestine and skin [27,50] As depicted in Fig. 4, the journey of OOC has provided great opportunity for innovation and novel solutions. It has been a decade since multiple well-developed models have been commercialized. Initial collaboration between National Center for Advancing Translational Science (NCATS) USA, the USFDA, and the US Defense Advanced Research Projects Agency (DARPA) in 2017 to develop organ on a chip for screening drug safety was followed by a collaborative study in 2018 between (NIH) and center for advancement in sciences in space in partnership with NASA to understand the role of microgravity on human health and disease [51]. Afterwards, several companies followed to commercialize tissue chip technology, with companies like *Emulate* pioneering lungs-on-chip, gut-on-chip, and blood-brain-barrier-on-chip systems in the US, while in Europe *Mimetas*, *Elvesys*, *AlveoliX* commercialized various organ-on-chip technologies. Companies like *TissUse* offer multi-organ chip systems for simultaneous cultivation of organs on a common microfluidic system while *BiomimX* offer generation of specialized predictive models of human organs and training cells to generate functional miniaturized human organs [51].

The commercialization of tissue chips is an important issue, as it reflects the difficulty of translating this technology from discovery to adoption and wide availability. Their goals include developing accurate cellular and organ microsystems for drug screening to reduce time and costs in drug development, as well as supporting innovative methods for faster treatments. Templates for the program and consortium have been established, outlining milestones and success criteria to guide initiatives and partnerships, highlighting plenty of opportunities that continue to exist and demand for further commercialization success stories [[52], [53], [54], [55]].

In addition to single-organ-on-chip, commercialized multi-organ-on-a-chip and human body-on-a-chip could potentially propel current technologies towards a huge leap in healthcare research [6].

### 3.1 Radiobiology

One of the imminent and emerging application sectors for organ chips is the study of internal radiotherapy in humans, which faces many challenges due to the lack of appropriate model for research and risk estimation [56].

The reaction of cells to radiation exposure is intricate, extending beyond the nucleus to encompass interactions among cells and their entire components. Therefore, models are essential for studying these mechanisms, requiring both accurate biological representation and user-friendly interfaces capable of simulating irradiation effectively [57].

Although there is limited exposure of radiations to human life, it is critical to understand their bio-effects. Radiotherapy could prove beneficial to patients as it destroys the cancer cells by the action of release of energetic photons, ions and electrons but has side-effects too such as swelling, low blood counts, skin irritation, heart complications, etc. [13,57] It is believed that as organ chips has a great success in pharmacological studies, it would also have a transformation in the field of radiobiology, particularly by enabling the development of protective therapies after radiation exposure and by stimulating the optimization of personal radiotherapy [6,50,56].

Moreover, tissue chip technology has the potential to accelerate *in-vitro* cancer research and bridge the gap between traditional *in-vitro* cell culture and *in-vivo* experiments [[56], [57], [58]]Studies show that as of today, scientists are more inclined towards testing of radiotherapy on cells that are cultured in the 2D environment on glass slides [56,57] However, attempts are made in the advancement in the OOCs technology in order to build the trust on the innovation by reducing the time consumption and investment. Among the important findings, it was found that endothelial cells grown in the typical 2D culture have notable amount of radiosensitivity than the cells present in the 3D vascular network [58]. This is significant in order to preserve healthy tissues while balancing radiation effects on tumour tissues. For this purpose, a basic understanding of the physiology of the cells in blood vessels within the organs in response to the radiotherapy needs to be taken into account [57,58]. With the advent of technologies such as 3D cell culture, tissue engineering, and microfluidics, it is now possible to stimulate the critical structures of the *in-vivo* tumour microenvironment and observe the functional characteristics of those structures [58] Compared to traditional pre-clinical models, it offers more realistic and accurate predictions of metastasis, tumour distribution, and growth mechanisms, as well as drug toxicity and therapeutic effects [56,58,59] This is supported by a study done on bone marrow-on-a-chip that found that the OOC system effectively mimicked the *in-vivo* response of living bone marrow to radiation countermeasure drugs while the conventional static bone marrow cultures failed to do so [60].

Similar studies have found that compared to 2D cell culturing, OOC systems allow the controlled co-culture of different cells to mimic various structures and functions of tissues and organs, such as blood–brain barrier, lung and heart [[61], [62], [63]].

Thus, OOC technology using microfluidic systems that helps in gathering crucial data of low-dose radiation that may kill some essential cells inducing severe diseases in embryos, which is currently not detectable. However with the help of OOC, early embryos and stem cell-derived embryonic tissues can be cultured and radiation disease can be studied [64,65].

### 3.2 Drug discovery and screening

COVID-pandemic has forced the government, researchers, and scientists to take special supervision and management regarding healthcare of their citizens [6,66] Organ-on-chip models, such as lung chips or intestinal chips, were proposed to evaluate SARS-CoV2 infected on the organ and test the efficacy of different drugs. For example, infected lung and colonic organoids derived from human pluripotent stem cells (hPSC) have been used to screen FDA-approved drugs and identify those that can inhibit SARS-CoV-2 infection. Chen and colleagues using hPSC derived lung organoids revealed that alveolar type II-like (AT2) cells were permissive to SARS-CoV2 infection and virus-induced chemokines and cytokines production, which was in accordance with clinical findings involving immune response in COVID- 19 patients. The drugs tested on OOCs include Remdesivir and Toremiphene these studies have used OOC models to test the effectiveness of drugs like Tocilizumab and Amodiaquine as potential viral entry inhibitors. These drugs have been evaluated for their efficacy in inhibiting viral replication, reducing inflammatory response, and potential viral entry inhibition in the context of SARS-CoV2 infection [[66], [67], [68]].

Since the development of the organ chips, it has been used by more and more pharmaceutical companies in order to discover new drugs and to test toxicity [56] Lack of effective drugs is the biggest threat in the treatment of disease caused by microorganisms hence drug discovery is an important aspect to combat microbial infection. A drug's development involves several stages, from the discovery of targets to lead optimization and preclinical testing to approval for clinical use [13,56]. *In-vitro* testing of drugs efficacy is not enough for application in patients, they have to be tested in animal models first. Therefore, *in-vivo* models are essential to investigate the use and efficacy of tested drugs [69,70] In the scientific community, there is general agreement that the current models used to test new diagnostic or treatment techniques do not adequately reflect the microenvironment found in humans [26] In many fields, including toxicology, pharmaceutical development, and cosmetic development that rely on animal testing and clinical trials, the development of micro-electromechanical biochips that replicate complex organ-level pathological responses can revolutionize the field [27,50,71] There is an ongoing experimental development of skin-on-a-chip for evaluating the effect of various makeup or cosmetic products [6] It can also be used to model whether the contained ingredients are actually safe to apply on skin [6,27] Since these platforms show a high degree of resemblance to crucial architectures and functionalities of human organs, stakeholders in the drug development process are increasingly interested in OOC applications [72].

Clinical trials are mainly done on middle age group people and females, but children are way too sensitive than adults, they might respond differently on exposure to drugs that are tested for adults [1,6]. Thus, diversification in population and genetic material can lead to risk of having an adverse reaction for which new technique needs to be adopted. This can be fulfilled by the utilization of MPSplatforms or tissue chips from which population on a chip can also be developed [66] The MPSsystem is intended to provide cell culture environments that are more accurate and reliable, closely matching the *in-vivo* processes of absorption, distribution, metabolism, and excretion (ADME) processes that occur in the human body [26,73] In the last decade, MPSplatforms have been increasingly used to support the ADME sciences, dedicated to reduce or eliminate regulatory pressure to move away from traditional animal safety assessment studies, with industries showing desire to develop methods for screening and characterizing drugs [73].

A common goal of MPS developers and users is to mimic a physiologically relevant part of an organ so that toxins and endogenous compounds can be tested and understood. The MPS revealed a degree of potential in drug safety assessment and has a possibility to reduce or replace the use of animals in safety assessment studies [18,74] Following this concept, patient-derived tumour cells, brain tissue matrix, and vascular endothelial cells were utilized to develop Glioblastoma-on-a-chip that replicated key clinical features with high accuracy, including resistance to chemoradiation and TMZ treatment. Cisplatin, KU60019 and a DNA repair inhibitor treatment were tested on two of the cell types, which resulted in reductions of cancer cells [75] In another study, Sobrino et al. also developed a tumour microenvironment and surrounding vasculature mimicking tumour-on-a-chip device. Culturing breast and colorectal cancer cells, they observed positive tumour regression using standard drug treatments. Apatinib and Vandetanib did not affect the vasculature, while a reduction in blood vessels due to targeting of Vascular Endothelial Growth Factor and other receptors by Linifanib and Cabozantinib [76] Similarly, a microfluidic vascular micro-organs development platform was devised by Phan et al. for effectiveness evaluation of cancer drugs against fighting cancer and inhibiting angiogenesis [77].

However, the construction of such mimicking chips is quite complex and its further coupling into various other organ chips is even a bigger challenge. One such example comes from Shuler group where they assessed the toxicity and mechanism of action of the anticancer drug 5-fluorouracil via Pharmacodynamics (PD) and Pharmacokinetics (PK) study using a 3D tumour–liver–bone marrow multi-organ chip system. They found the liver compartment to be more resistant towards the drug than the bone marrow and tumor compartments [78,79].

Along similar lines, Herald et al. assessed anticancer drug nicotine and Cisplatin by developing gut–liver–kidney and bone marrow–liver–kidney multi-organ systems to estimate the PK parameters via oral and intravenous administrations respectively. Cisplatin did not express any hepatotoxicity but express myeloid toxicity and nephrotoxicity in bone marrow–liver–kidney multi organ system, which was suggested to help drug optimization for initial clinical trials [67].

Among the major challenges such as, organ-specific flow rates, maintaining sterility conditions, incorporation of immune cells and other cell types, there is a limitation in ADME studies [74]. This is important due to the fact that major failures that are experienced in drug development ultimately resulting in its withdrawal are due to liver metabolism and related toxicity [18,71,80] In the human body, drugs are metabolized by the liver and the resulting compounds may have the chances to cause unexpected toxicity to off-target organs, such as the heart [49,81] Therefore, it is essential for the drug developers to decipher the interactions generated between the liver and the administered drugs during pre-clinical testing in order to design and develop optimal drugs [72,82] Besides this, the primary cause of drug failure is cardiotoxicity, a condition that arises due to blood which cannot be properly pumped via the heart because of alterations in heart kinetics [81,83] Cardiotoxicity testing performed *in-vitro* is conducted primarily using cell lines expressing cardiac specific ion channels, using which the drug's interaction with these channels can be monitored directly [73,81] However, challenges are faced till now to meet accurate, more efficient and reliable methods for drug screening. By using miniature systems that represent the complex structure and function of the heart and liver, organ chips (liver-cardiovascular chip models) aim to overcome those challenges and limitations of hepatic and cardiovascular toxicity tests and drug screening methods which use less complicated *in-vitro* platforms [18,81,83].

### 3.3 Big leap of Research in space

**Fig. 5:**
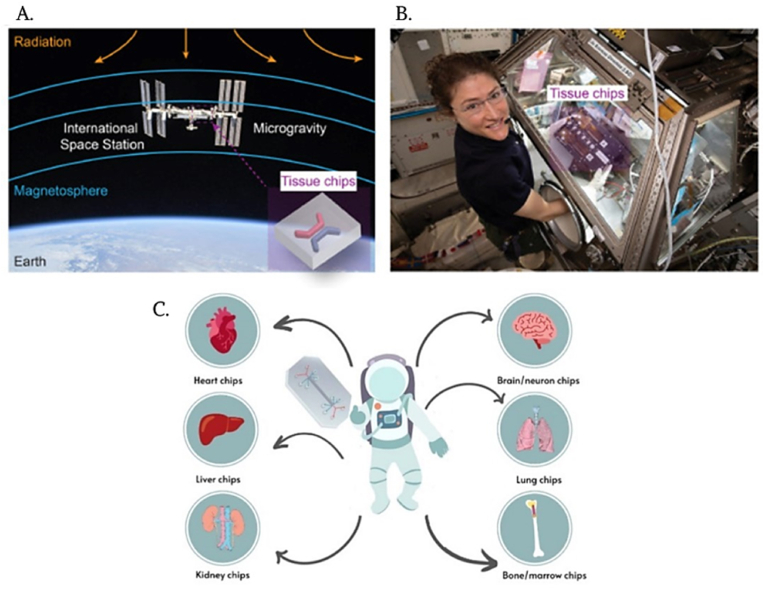
(A) Potential use of tissue chips for studying the biological effects of microgravity and radiation in spaceflight environments. Photograph credit (iss056e201352): NASA/Roscosmos. (B) A kidney chip operated by astronaut Christina Koch on board the International Space Station (ISS). A reduction in footprint and improved automation of liquid handling makes tissue chips appealing. (C) A description of tissue chips for resembling several physiological functions at the tissue level. Illustration of multiple tissues that can be integrated into one chip *Adapted with permission from ref* [86].

The Tissue Chips in Space initiative seeks to better understand the role of microgravity on human health and disease and to translate that understanding to improved human health on Earth. Space travel or spaceflight cause many significant changes in the human body. The same behaviour is predicted with the tissue chips like an astronaut's body, experiencing the identical quite rapid change [84] In microgravity, changes occur in human health and human cells that resemble accelerated aging and disease processes which implies space related changes occur much faster that may enable scientists to use MPS in space to model changes that might take months or years to happen on earth [84] This research may also help scientists and researchers advance the utilization of tissue chip technologies for more efficient pharmaceutical testing on Earth, and could be used for understanding how diseases develop in healthy tissues. Translational research at the ISS National Lab have been involved in providing revolutionary opportunities to study these effects of a microgravity environment on the human body [85] It will also contribute in understanding the process of aging and could reveal molecular targets that slow down these processes. Space Shuttle missions have been circumscribed to the analysis of attainable biological samples including blood and urine samples, preventing any examinations at cellular or molecular level of organs or tissues [84]It might also be useful for other investigators to conduct experiments in microgravity for the purpose of developing complex tissues formed from many kinds of cells (Fig. 5C) [86] There are two important factors that affect cell and tissue culture in microgravity: buoyancy and density-driven convection. Essentially, these factors cause cells to aggregate based on their size and density and sink to the bottom of culture vessels instead of distributing as they should in anatomically correct vessels [86]Conversely, cells in microgravity and high energy radiation (Fig. 5A) tend to self-assemble slowly and aggregate based on cell-to-cell contact and their physiologic affinity [86].Thus, with the combination of 3D microfluidic tissue chips and microgravity environments, insights into disease models and therapeutic targets can be gained in a unique way (Fig. 5B). It has been observed that as people get older, their immune response is compromised and gradually becomes weakened [86,87]In order to examine this relationship between aging, immune response and healing outcomes, space station is being launched for which it is believed that the process may be accelerated in microgravity [84] By looking at immune function in microgravity, the research team will study aging biology, and then after returning to Earth, the research team will study the ability of cells to recover [84,87].

### 3.4 Experimental regenerative medicines and disease modelling

Fully repairing or regenerating damaged tissues or organs and restoring their functions has been a goal of for ages now. The approach of tissue engineering and regenerative medicine appears to make it possible [88]. Regenerative medicine is the branch of medicine that develops methods to regrow, repair or replace damaged or diseased cells, organs, or tissues while also incorporating the self-healing power [84].

Regenerative medicine includes the generation and use of therapeutic stem cells, tissue engineering and also the assembly of artificial organs [23,66] The emergence of microfluidics and therefore the development of novel microfluidic devices such as tissue chips, have been able to give an upliftment to regenerative medicine research [89] This field deals with both providing an understanding of the causes of the damage to the tissues as well as methods for restoring the functionality of the tissue and the mechanisms by which stem cells repair the tissue [90]. One such microfluidic system, a liver-on-chip device, was developed by Bavli et al. with integrated sensors for monitoring of mitochondrial dysfunction which was relevant clinically for chemical and pharmaceutical toxicity analysis. Real-time analysis of mitochondrial respiration and stress was possible using automated microfluidic techniques with replaceable medical-grade sensors. Such studies revealed that mitochondrial dysfunction can occur even at previously deemed safe drug concentrations. Similarly, development of a cardiac living patch for treating heart attacks proved useful towards monitoring cellular electrical characteristics and regulation of tissue function by modulating cell contraction [[91], [92], [93], [94]].

Targeted differentiation of stem cells has created high expectation on the regeneration of organs [56] This concept was further strengthened as laboratory-derived human fibroblast/hepatocyte cellular sheets were implanted mice skin for liver tissue engineering subcutaneously which promoted fibroblasts and primary hepatocytes contact while retaining the phenotype and function of primary hepatocytes, such as ALB and alpha 1-*anti*-trypsin. Significant formation of vascular networks was also observed, untimely highlighting the potential of using liver organoids to regenerate liver cells resembling native liver tissue [95]Another successful example of Targeted differentiation using stem cells was revealed via a study with heart-on-a-chip platform mimicking natural mechanical conditions experienced by heart cells, developed using stem cell-derived cardiomyocytes from rats and humans. Mature and functional micro-engineered cardiac tissues was successfully developed by exposure to controlled strains in a specific direction to simulate the heart's movements [96].

The organ chip technology provides a broad platform to understand key aspects of how various fabrication strategies affect cell viability and tissue function based on regenerative medicines [56].

**Fig. 6:**
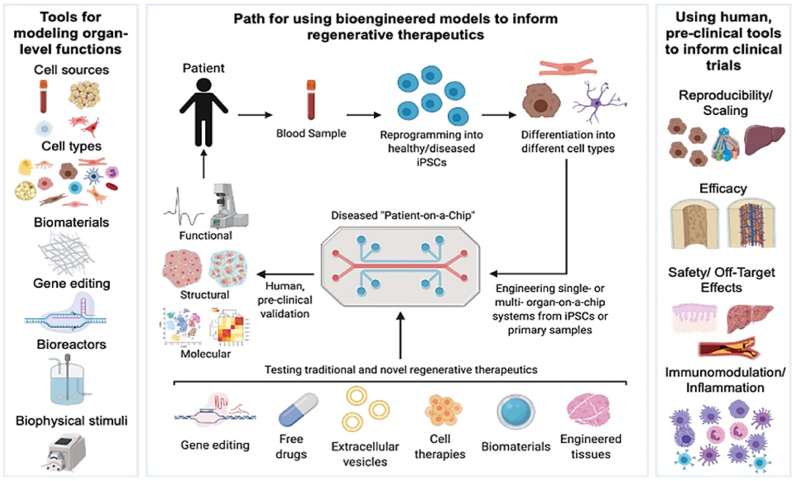
Novel *in-vitro* human model showing possible configurations for experimental regenerative medicines and disease modelling. *Adapted with permission from ref.* [97].

In the past few decades, regenerative medicine has provided evidence that technologies like decellularization, 3D bioprinting, cell and organ engineering, and blastocyst complementation may offer platforms for the bioengineering, repair, and regeneration of transplantable organs [10,20,97] Bioengineered models *in vitro* in combination with animal models are now being frequently being used to test innovative regenerative medicine techniques (Fig. 6) [97]Although emerging data is promising, the complexity of solid organs poses a significant challenge, and further research and substantial investments are needed [97]Advancements in basic research knowledge of stem cells, biomaterial, and developmental biology in combination with various biotechnologies and bioreactors are crucial for fast growth [97,98] Development and incorporation of chip technologies, organoids, and 3D bioprinters are opening new avenues and directions for the future [10,13,98].

### 3.5 Precision healthcare/individual susceptibility

**Fig. 7:**
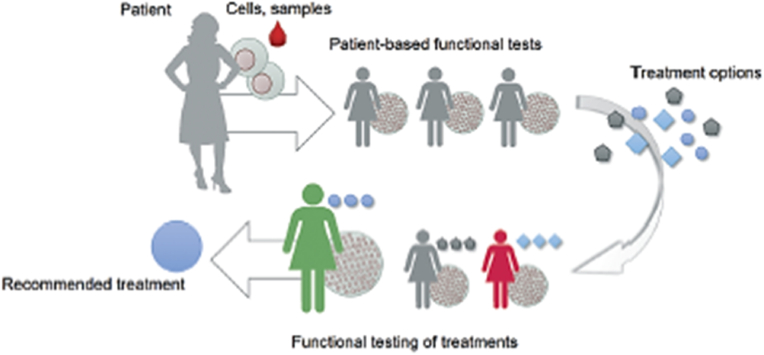
Functional tests are useful in selecting, optimizing, or developing treatments. Patient material-based tests provide a functional readout associated with patient outcomes. *Adapted with permission from* [21].

Huge effort has been made to evolve the use of tissue chips to reflect individual physiology which is its principal role in personalised drug development. MPS platforms could be useful tools to model tissues from individual patients for therapeutic analysis and for precision medicine efforts. Deaths of patients due to unavailability of proper drug therapy for lethal and rare diseases have raised awareness towards the discovery of drugs for their treatment. Also, tissue chips offer the possibility to model population-wide variation, enabling genetic factors such as gender, as well as environmental factors such as exposure to infectious agents or toxins, to be modelled [26]The main aim of personalised medicine is not to simply treat the disease for an individual but also to decide the best possible cure and treatment. A comprehensive approach to healthcare which involves reproducing key features of the structure and function of the patient tissues *in-vitro* in a controlled manner, capturing the interaction between heterotypic cells, followed by assessing how these *in-vitro* models react to novel treatments. This process is referred to as ‘functional testing’ (Fig. 7) [21] In the most advanced functional tests currently performed, patient stem cell-derived three-dimensional spheroids or organoids are used, which capture important structural attributes of the tissues of the patient [21,99] A increasing identification of number of groups of non-responders responsible for ineffectiveness of certain drugs is making precision medicine a relevant concept in medicine, toxicology, pharmacology and biomedical science as a way of promoting, maintaining and restoring health [20,50,99] Precision medicine offers treatment customised or personalised to each individual.

Remarkable examples include a lung-on-a-chip with breathing mimicking mechanism of expanding and contracting, blood vessels-on-chips replicating the circulation of metastatic tumour cells, a gut-on-a-chip simulating intestinal muscles movement and microbe flow, a multi-organ chip with liver and pancreas spheroids that dynamically maintains glucose balance, and engineered neuromuscular junctions that are combined with contracting muscle tissu [21,100,101].

Furthermore, proof-of-concept studies have successfully demonstrated co-culturing of intestinal bacteria like *E. coli* and *L. rhamnosus* in a state of dynamic equilibrium with intestinal epithelium within organs on-chips [21]Successful reports have shown that tissue chip models are more effective than rodent or animal models in determining drug safety and its effectiveness, allowing scientists and drug developers to develop individualized strategies for disease prevention and treatment [21,99] This new technology has enabled many researchers and drug developers to take advantage of this new opportunity [21,102]. Implementation of personalised organ-on-chips in precision medicines is however, challenged due to the requirement for access to personal samples and corresponding health data to have a meaningful predictive outcome [21,99,102].

### 3.6 Therapy development

**Fig. 8:**
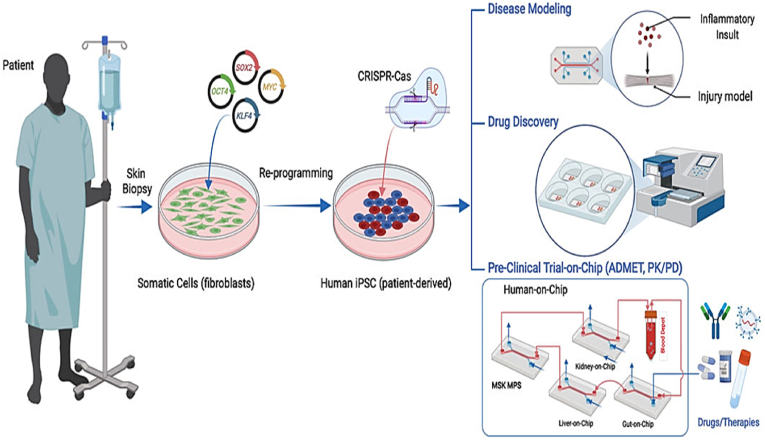
Depiction of Human induced pluripotent stem cells (hiPSCs) applications in MPS platforms including disease modelling, drug discovery, and pre-Clinical trial-on-Chip, including ADMET (absorbance, distribution, metabolism, excretion, and toxicity). *Adapted with permission from ref.* [71].

Developing and supervising experimentations are extremely difficult for rare and lethal diseases thus, adapting tissue chips or MPS platform for rare disease therapy is very crucial in assuring the treatments are available and feasible to medicate severe diseases [103] One of the prime roles of this technology is speeding up the research and also this enables to test the effect of a wide range of drug concentration on the efficiency of the medicine [49] During the course of drug development, the first round of tests can be conducted many times without risking financial complexities [49] Being a human cell-based approach, tissue chips facilitate scientists and researchers to predict more accurately which drug will prove beneficial and which one will be toxic, allowing them to understand their efficacy rate [1] This would provide a positive outlook towards the developing pharmaceutical companies and a hope for people [2] A growing number of *in-vitro* models of the vasculature have been developed using human induced pluripotent stem cells derived from healthy individuals and patients [23,104] High-efficiency human endothelial cells (ECs), pericytes, and vascular smooth muscle cells (VSMCs) can now be generated from hiPSCs and used as a basis for *in-vitro* blood vessel models using microfluidic chips [104,105]As a recent advance in biomedical science, the integration of major tissues and organs in the human body on a single chip or connected chips has enabled the prediction of safety, efficacy, and PK/PD of drug candidates in humans (Fig. 8) [71] Researchers have shown that adding living renal proximal tubular cells to microchannels or hollow fibres improves waste excretion, glucose reabsorption, and clearing of middle-sized molecules [71,74]. Recently an elementary “human-on-a-chip” was reported to provide a possibility to mimic the internal niche of human body *in-vitro*. On similar lines the first “organ-on-a-chip” of the alveolar capillary interface was built, reconstructing the lung function with cyclic mechanical stress and strains [56] In addition, multi-organ-on-chip has been developed that consider inter-organ responses to obtain more accurate therapeutic reaction highlighting the unique information that is not accessible using single OOC [56,106] Throughput, analytical, and quantitation capabilities have been significantly increased as OOC technology has been industrialized; this has been a continuous demand from the pharmaceutical and biotechnological industries, which are seeking new and improved screening methods to overcome the high attrition rates in drug discovery and in turn in therapy development [71,97,103,105].

### 3.7 Tissue chips as 3-D printing model

**Fig. 9:**
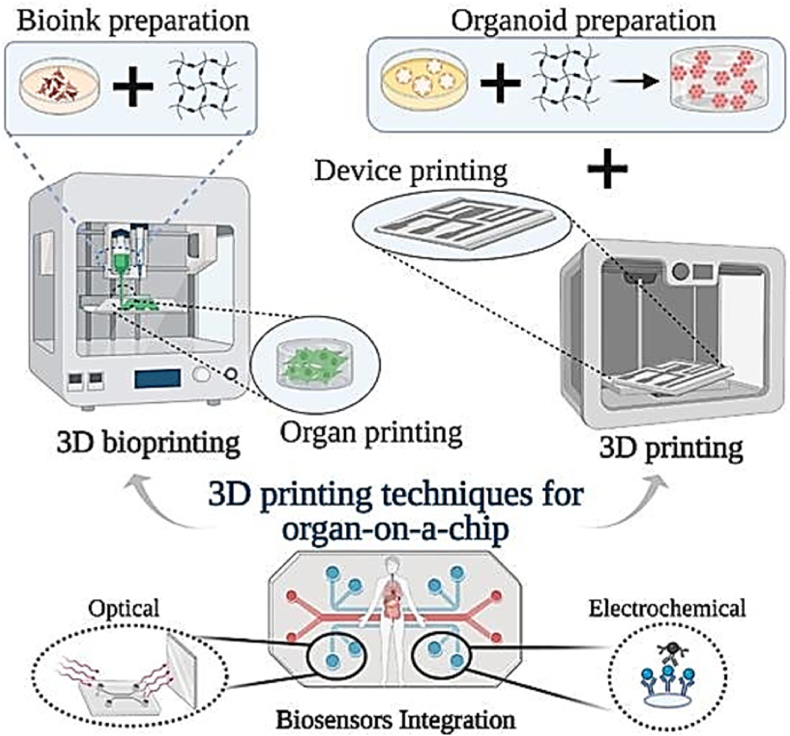
Illustration of the progress of organ-on-a-chip platforms as achieved by 3D printing techniques. *Adapted with permission from ref.* [109].

While OOCs were initially much more fundamental and lacking in necessary adjustability, today they are incredibly advanced as scientists move closer and closer to their goal of being able to transplant 3D printed organs into the human body with success. Although bioprinting has progressed immensely, the technique is still loaded with challenges due to the delicate nature of tissue engineering [107] Organ-on-a-chip engineering desires to create artificial living organs that mimic the complex and physiological responses of real organs, with the purpose to test drugs by precisely operating the cells and their ambience [108]As a first step towards the achievement of highly reliable 3D culture models for diseases, drug development and testing, as well as drug delivery, selecting the appropriate fabrication technique is crucial [109,110] Experiencing more substantial outcome of this kind of chip, pharmaceutical companies will potentially be able to measure direct outputs needed [27,108]For instance, the delivery of biochemical substances would be screened to confirm that even though it may benefit one cell type, it does not compromise the functions of others [27]It is probably already possible to print these organs with 3D printers to demonstrate the ability of this technique to achieve the physiological relevance and can be applied to drug screening [27,108]. 3D bioprinting/printing techniques are becoming increasingly applicable to the manufacturing of OOC platforms [109] Incorporating 3D printing techniques along with the production of OOC broadens up the possibility of replicating the *in-vivo* environment by creating heterogeneous structures utilizing different cell types at the same time (Fig. 9) [109] These techniques include embedded extrusion bioprinting, direct Laser Writing (DLW) and direct Laser Interference Patterning (DLIP), stereolithography (SLA) and bioprinting. Among the most popular bioprinting is the embedded extrusion bioprinting which is suitable for a broad range of biocompatible materials, some of which are mentioned here (Table 1) [108,110] These techniques involve the process of deposition of biological material in a layer-by-layer fashion which proposes the use of different types of needles to create 3D structures like tissues and organs. Though this approach has great potential to create 3D organs or tissues, the approach has experienced advancement in order to improve fabrication of the models manufactured [109,110].

**Table 1:** Examples of developed OOC platforms with their respective printing techniques.

OOC Platform	Cell Type	Method of Printing	Bioink	Schematic representation	Source
Nervous System-on-a-Chip	Epithelial cells, Superior cervical ganglia, Schwann cells and Hippocampal neurons	Micro-extrusion 3D printing strategies	–		Adapted with permission from Ref. [109]
Liver Fibrosis-on-a chip	Hepatic Stellate cells, HepaRG and HUVECs	Microextrusion bioprinting	Gelatin and liver dECM bioinks primarily of type 1 collagen		Adapted with permission from Ref: [111]
Vessel-like structures-on-a-chip	L929 fibroblasts, endothelial cells and smooth muscle cells	Coaxial nozzle-assisted extrusion-based bioprinting	Cell-laden alginate filaments		Adapted with permission from Ref. [112]
Gut-on-a-chip	HUVECs and Caco-2 cells	Dual cell-printing system supplemented with a core-shell nozzle	Cell-laden collagen bioinks		Adapted with permission from Ref. [113]
Placenta-on-a-chip	hMSCs and Human placental cell line	Extrusion-based 3D bi82oprinting	GelMA		Adapted with permission from Ref. [114]

## 4 Tissue chips as an advancement technology

The prime advantage of OOC technology is imparting the potential to build a specific human model that has the power to indicate the functional responses on the extent of tissues or organ when cells are made to experience effective environment as that within a human body so that they can mimic their natural niche *in-vivo*, thereby evading the use of inefficient animal models [56]The inadequate understanding of human physiology and biology and also the limited potential for experimentation has stressed the urge for innovative *in-vitro* models. Ideally, such a system would enable the scientists and researchers to accurately recapitulate disease aetiology and progression, predict responses to treatment, and are being used to identify species-specific toxicities. Preclinical drug testing has traditionally relied on animal models, but cell-based assays are proving to be more trustworthy.

Tissue chips are handy and reliable which is its key potential that has allowed many engineers and scientists to operate and develop this innovation [27]It has the potential and enable the insights of the biology driving mechanisms of multiple diseases such as neurodegenerative diseases.

Automated instruments have been developed that tend to give the engineering controls that are required to link and interconnect ten or more organ chips together with the benchmark for fluid control. It creates an easy user interface that is automated and control fluid flow [1] Despite having such potential, the traditional cell culture system is still used widely and very few studies have focussed on this innovation [56].

Translational Organ-on-chip Platform (TOP) has been introduced where controlling and analysis of organ chips are standardized without compromising user and developer flexibility. It has made it easier to control the fluidics from, to and within the chips by providing infrastructure to make OOCs plug and play [6]This platform provides an adherence to the design rule without the complexities drawn due to microfluidics and thus play an adaptive role for the biologists who can access the biological protocols automatically with setting the parameters and hit click of a button [30].

It encompasses various types of fluidic control boards (FCBs) and microfluidic biochip modules, including advanced FCBs with pneumatic control and simpler FCBs for medium recirculation between two fluid reservoir modules. This platform also includes a readout and control system tailored to OOC research and is being supported by a network of stakeholders from academia and industry. TOP is being utilized in several ongoing research projects, such as gut-on-a-chip, heart-on-a-chip, and cancer-on-a-chip models. While current development focuses on modules and interconnects, future efforts will expand to include control systems and software [115].

Various experimentation models of TOP to see effects of mixture of drugs are being developed, like Heart chips with a platform mixing device and two heart-on-chip-devices with integrated force sensors [6]. With the help of a software, two chips are selected and for each chip different mixing protocols is being adjusted and then, the correct mixture is routed at the right time to the right model automatically [116].

## 5 Challenges and obstacles in the pathway

Tissue chip platforms aim to translate basic discoveries into clinical applications but face biological and technical obstacles [26] Integrating multiple chips shows promise for drug toxicity screening, yet hurdles remain [26,99] Limited individualized use restricts broader application for observing specific organ functions. While developed to understand overall organ responses, miniaturizing and linking tissue architectures poses significant challenges [54] Recent advances are made in constructing Human-on-a-chip or Multi-organ-on-a-chip looking such complexities but rather much more is needed for accuracy [6,54].

### 5.1 Biological challenges

One key focus involved in its biology is appropriate organ scaling and cell organizing. Though, coupling of platforms results in proper understanding *in-vivo* physiology of humans, it is still a critical consideration when platform systems are linked physically [26] Every human being has different body structure and so their immune responses which helps them fight diseases. As immune mechanisms can change the response to drugs, the inclusion of immune components in tissue chip platforms could prove critical in the near future [26]Tissue chip model as discussed above could be a powerful tool for precision medicine efforts considering the simulation of population distinction [117].

Physiological responses which depend on many factors such as age, genomes, immune capabilities and environment, vary a lot within the population thus making designing and manufacturing tissue on chip platforms more challenging [26]Moreover, to make cells and tissues experience their natural niche, proper vascularization should be provided which requires blood mimetic or universal medium. Apart from these, the consideration of circadian cycles of cells and tissues is also vital. Considering all these biological factors, together makes it a complex issue to deal [54].

#### 5.1.1 Organ scaling

As such tissue on chip platforms pose numerous biological challenges, organ scaling is crucial to ensuring their physiological relevance to that of organs *in-vivo*. The size and dimensions of the organs i.e., organ scaling and also cell numbers, needs to be relatively appropriate for observing the proper functioning of such organs *in-vitro* [26] Allometric scaling can be used to quantify the relationship between organs of different sizes in OOCs as compared to *in-vivo* tissues. Yet this method finds limited efficiency when functional complexities of human tissues are considered [26] For more realistic determination of organ masses, functional scaling may be used where preservation of tissue function gains priority, forming as an appropriate strategy to regulate organ masses with suitable proportions of active cells between platforms to show accurate bodily or physiologically relevant responses in designed OOC systems [26,54]. This approach allows maintenance of organ-specific functions at appropriate relative magnitudes. In order to create microscale analogues of humans, the micro proportions of the organs that need to be scaled down has to be taken into account due to the fact that precise readings and findings are dependent on these dimensions and size [118] Marking its vitality, if suppose the dimensions of two organs linked in chips outside the natural niche is different from that of the actual dimensions of organs *in-vivo*, the readouts of the metabolite would not be appropriate [26,54] To overcome this problem, allometric scaling and functional scaling can be used. As per data, allometric scaling is a tool used by drug developers use to predict human pharmacokinetic based on animal data considering the relationship of body size, shape, anatomy, and physiology. An organ of different sizes can be compared using this technique [26,118] It is, however, important to realize that simple scales do not necessarily work when comparing human *in-vivo* tissues with MPS because of their complexity and size differentials [26]In order to determine meaningful ratios between organ masses, functional scaling could be a more appropriate strategy, since it takes into account the tissue's function.

#### 5.1.2 Linking platform systems

Modelling human disease pathologies with tissue chips holds much promise for use in the development and screening of drugs and therapeutics [54] However, one of the major challenges is creating micro platforms for studies, which involves creating air-liquid interfaces, mechanical and physiological stresses, and implementing barrier functions that affect the delivery of drugs and chemicals across the endothelial barrier [54,119] Currently, OOC designs, manufacturing, and operating procedures are not standardized, so end users must invest time and resources in setting up the systems and customizing their testing protocols [6]. Engineered systems still lack the ability to fully recreate the vessels, tubes, and ducts in human tissue and organs [8,118].

#### 5.1.3 Complementing of immune system components

Despite providing the MPSs with vascularization to experience the natural environment, another challenge arises in the presence of immune components since the body's functions rely on hormonal feedback loops and regulatory mechanisms [120,121]. The research and studies suggest that better inventions are required in order to overcome this challenge of implementing immune components in the MPSs or tissue chip models which would provide the better and accurate functioning of the system [121,122] The natural physiological body system in humans continuously communicate and coordinate with each other through various means, by producing essential microbial metabolites which is aided by immune responses. This is also dependent on genetic heterogeneity which is responsible for various chemical and biological parameters in the organisms [71,121] Consequently, natural niche is manipulated genetically, chemically, or immunologically, as well as etiological factors, the metabolism of microbial drugs, the genetic heterogeneity, and pharmacological validation which are currently difficult to integrate into microphysiological systems [120,123] This integration of immune components could prove to be a great benefit for the emergence of precision medicines.

#### 5.1.4 Vascularization for natural niche

A major challenge of integrating multiple tissue chips is establishing communication between them that accurately mirrors the crosstalk experienced *in-vivo* [99]. It is imperative that multiple organs work together in order to ensure the human body functions properly physiologically. In *in-vivo* systems, organs are physically separated which is mediated by the vascular system which consists of blood and lymph circulation to maintain overall viability and homeostasis [99]. Oxygen exchange and mass transfer are the defining factors for tissue and organ survival, which are facilitated by the circulatory system. The cells seeded on OOC also need a continuous supply of appropriate nutrients for proper functioning, OOCs must incorporate a circulatory system to simulate tissue microenvironments and physiological functions [34]. Different research groups are working on this situation and are investigating for a blood mimetic agent to address this problem [6,54] Researchers are also continuously working to overcome the obstacles associated with the field of vascular tissue engineering [35] A number of studies have shown that 3D bio-printing is a promising technology for the development of this technique, but more evidences need to be collected and more research needs to be conducted [8] The complexity in the development of the constructs is till now challenging [8].

### 5.2 Technical challenges

Tissue chips designed in complex and miniaturized fashion itself describes its difficulty level. Technically, a number of challenges can be confronted for coupling the MPS platforms physically [54] Interconnecting or linking of Tissue chips to form MPS platforms have possibilities to cause the formation of bubbles in the platform that could not only hinder the fluid flow between the cells and tissues more than in larger systems but can also cause a loss of sterility within systems [26] Additionally, sterilisation and disinfection of every equipment and platforms needs a great count while connecting such devices, initiating from the implanting of cells till when they get matured independently and further, to avoid any unsuitable outcomes [26,54].

Constructing tissue chips and MPS platform requires careful consideration of material selection and fabrication techniques to resolve biocompatibility issues and to facilitate easy monitoring and detection [26]. These devices require biocompatible materials in order to provide structural support for engineered tissues and to promote tissues with either healthy or diseased traits or function [26,119]. Manufacturers of medical devices, researchers and scientists that are involved in contemporary drug delivery technologies, face a significant challenge for the biocompatibility of these devices [119] Properties that the desirable biomaterial includes are transparency to facilitate on-chip imaging, negligible cellular toxicity, ensuring adequate oxygen supply to the cells in culture with the aid of sufficient gas permeability and minimal absorption of drugs, chemicals and biomolecules for accurate results [26,54,83] PDMS is a polymer widely used for fabrication and prototyping of microfluidic chips [124] Its usage has allowed the much needed shrinkage in size of tissue chips. It is transparent and most importantly, has low auto-fluorescence which means it has reduced natural emission of light that is helpful to observe certain cell molecules as they get less exited when exposed to UV or visible radiation. PDMS is the most widely employed material but comes with drawbacks as the resultant film is thicker than the actual *in-vivo* morphology [6,26]. Thus, alternative material needs to be explored for least difficulties. Most prominently, the complexity of observing organ functionality increases as the number of organ chip integrated increase which is currently unsolvable and needs a focus for further growth of the innovation [6,26].

#### 5.2.1 Sterility and bubble-free platforms

When connecting such devices, sterilising and disinfecting all devices needs a great deal of thought, starting from implanting the cells until when they mature independently, and onwards, to avoid any unintended consequences [125]In light of this, sterile preparation is extremely important in the healthcare industry. As a result of their use, systems and conditions must be sterile in order to prevent the possibility of microbial degradation or infection [123]It is essential for the platforms to nullify or make the systems bubble-free which often is the issue while linking platforms to construct multi-microphysiological systems [71,123]The bubble-free platforms omit or lessen the risk of time-lapsing between the fluid flow which may hinder in the process of drug discovery and various other researches including precision medicines. A bubble in such a platform can impede fluid flow to the cells, preventing accurate readings, which is why the fluid should be pumped to the cells as quickly as possible by actually maintaining the condition as there is in the natural niche of the cells [126].

#### 5.2.2 Platform-specific flow rate differences

The development of complex *in-vitro* models of vascular networks in hydrogels has been made possible by recent advances in microfluidic device design and fabrication methods [119,127]. Researchers are still facing issues with differences in flow rates according to platform taken into considerations [8,128]. For a more realistic mimic of native vasculature, more complex microfluidic designs replicate mechanical cues, such as shear flow and strain [30,129] Several mechanisms operate in conjunction with fluid flow in order to drive the function of tissues, including unidirectional flow that can act as a mechanobiological signal to the endothelial cells, and perfusion to maintain homeostasis in tissues by controlling levels of nutrients, oxygen, and wastes [[128], [129], [130]] It may be important to replicate tissue-like behaviour *in-vivo* in order to mimic fluid flow and relevant hemodynamic stresses, including pressure, shear, and stretch and thus, it is vital to consider different flow rate conditions in a certain type of organ when constructing an OOC. More accurate and low-cost alternatives to the current methods employed in therapeutic studies and disease modelling may be provided by the better characterization and development of the profound technique [8,129].

#### 5.2.3 Optimization of oxygen and nutrient levels

The vascular system is most prevalent organ in the human body which is a circulatory system of vessels that transports oxygen and nutrients to other bodily systems such as the respiratory system, digestive system, kidneys, and urine system [32,131]As a result, the vasculature serves an important and necessary role in keeping the body stable and safeguarding optimal organ function. Constructing the chips in a way that permits oxygen and nutrients to be distributed to cells according to requirements becomes essential [131]Different oxygenation levels needed to be controlled between different organ systems, or even within one organ system [32,36,80] As the cells in our body are supplied with adequate amount of oxygen and nutrients as per needs by the body, the same condition becomes essential to be followed in order to get accurate readings and better findings [36,122]. However, many factors influencing cellular needs and bodily needs which affects the requirements of the optimal levels of oxygen and nutrient supply makes it difficult for the technique developers to make the systems mimic the actual environment [71,132]Therefore, multi-organ systems may require sophisticated engineering, such as selectively oxygenating or deoxygenating local recirculation reservoirs to supply the appropriate tissues and organs [20,71].

## 6 Conclusion and future prospects

Advances in micro-engineering and biomedical field have led to the advent of OOC systems that is premeditated to recapitulate characteristics of living human organs. OOC is the convergence of two emerging research areas, microfluidics, and tissue engineering. The primary objective of these chips is to integrate numerous organs into a single chip and to further develop more complex multi-organ chip model, finally achieving the goal to simulate whole human body on a chip which would be a game-changing advancement in microengineering and drug discovery. Although OOC technology foresees enormous promises in the healthcare sector, it is yet to mark its footprints in the market.

Transforming biomedical research, OOCs have provided 3D platforms with appreciable biological representation of tissue morphologies and functions, expanding the scope for research and risk estimation through realistic and accurate microenvironments. Radiobiology has immensely benefitted from OOCs as successful radiotoxicity estimations and countermeasure drug studies have been conducted with organ, tumour and embryological tissues, paving way for faster, cheaper, and more accurate pharmacological studies. The recent COVID-pandemic saw drug discovery and drug screening against SARS-CoV-2 done with OOCs of specific target cells and organ tissues. Drug efficacy and ADME studies of various anticancer drugs on a diverse set of OOCs have sped up clinical trials, immensely benefiting public health and pharmaceutical industries. Tremors of such studies have further resonated with practical applications in personalised medicine and therapy development where rare and specific cases have been screened and monitored, providing data that was effective, efficient and critical for patient treatment formulation and administration. The vast applications of OOC systems expand horizons to unforeseen lengths as effects of microgravity on human physiology were successful carried out at the ISS National Lab with microgravity environments. Another such landmark was achieved when experimental regenerative medicine and disease modelling researches were carried out on OOC platforms. Targeted differentiation of stem cells for tissue regeneration and sensor incorporation for live monitoring of disease pathology accelerated the understanding of these complex phenomena by providing promising and realistic data. Creation of such complex living systems have been synchronous with improvement and development of 3D printing technologies, providing further insight into designing of highly reliable 3D cultures for disease modelling, drug development and testing, as well as drug delivery system testing. Incorporating 3D printing techniques along with the production of OOC will further broaden up the possibility of successfully replicating the *in-vivo* organ environment by creating heterogeneous structures utilizing different cell types at the same time via different approaches with potential to produce biocompatible materials.

It is important to understand that the biological elements and the engineering aspect are equally important for successfully bio-mimicking human tissues in *in-vitro* environment. Proper design of the microfluidic device for this purpose is of utmost importance where the micro-channels play a critical role to create tissue-specific structures. The pumps must be controlled to achieve a laminar fluid flow that can mimic an actual laminar and streamlined blood flow in the vesicles. The physical factors like mechanical, electrical, and chemical signals should be to be meticulously controlled in the microfluidic devices to precisely mimic the physiological and pathological micro-environment in cells. Overall, fabrication methods strongly affect the design and performance of an OOC. Integration of biosensors along with the OOCs is an intelligent move to initiate real-time-analysis as well as monitoring of tissue functionality and micro-environments.

It is well understood that mimicking the very complex microenvironment of tissues/organs onto micro-dimensional devices is very challenging. Incorporating smart biomaterials such as stimuli responsive biomaterials onto the OOCs may improve reconstruction of the cellular three-dimensional microenvironment. OOC technology can be of tremendous benefit in studying pathophysiology of highly infectious microbes such as SARS-CoV2. In this regard, the OOC technology may provide a breakthrough in fighting future pandemics.

As of current update and need, OOC technology necessitates technically skilled personnel those who are trained to handle micro-scale devices. The most likely reason of its unfamiliarity is its heavy user dependency thus causing low reproducibility. Automated robotics handled systems can open the path for high throughput manufacturing and improved user-friendliness.

## Notes

The authors declare no competing financial interest.

## CRediT authorship contribution statement

**Prerna Suchitan Modi:** Writing – original draft. **Abhishek Singh:** Writing – review & editing. **Awyang Chaturvedi:** Writing – review & editing. **Shailly Agarwal:** Writing – review & editing. **Raghav Dutta:** Writing – review & editing. **Ranu Nayak:** Conceptualization. **Alok Kumar Singh:** Conceptualization, Formal analysis, Supervision.
